# Screening eaves of houses reduces indoor mosquito density in rural, western Kenya

**DOI:** 10.1186/s12936-022-04397-y

**Published:** 2022-12-09

**Authors:** Bernard Abong’o, John E. Gimnig, Diana Omoke, Eric Ochomo, Edward D. Walker

**Affiliations:** 1 Centre for Global Health Research, Kenya Medical Research Institute, P.O. Box 1578-40100, Kisumu, Kenya; 2 Centers for Disease Control and Prevention, Division of Parasitic Diseases, Atlanta, GA 30341 USA; 3 Michigan State University, 6169 Biomedical Physical Sciences Building, East Lansing, MI 48824 USA

**Keywords:** Eave screening, *Anopheles funestus*, *Anopheles gambiae*

## Abstract

**Background:**

Despite the scale-up of insecticide-treated nets and indoor residual spraying, the bulk of malaria transmission in western Kenya still occurs indoors, late at night. House improvement is a potential long-term solution to further reduce malaria transmission in the region.

**Methods:**

The impact of eave screening on mosquito densities was evaluated in two rural villages in western Kenya. One-hundred-and-twenty pairs of structurally similar, neighbouring houses were used in the study. In each pair, one house was randomly selected to receive eave screening at the beginning of the study while the other remained unscreened until the end of the sampling period. Mosquito sampling was performed monthly by motorized aspiration method for 4 months. The collected mosquitoes were analysed for species identification.

**Results:**

Compared to unscreened houses, significantly fewer female *Anopheles funestus* (RR = 0.40, 95% CI 0.29–0.55), *Anopheles gambiae* Complex (RR = 0.46, 95% CI 0.34–0.62) and *Culex* species (RR = 0.53, 95% CI 0.45–0.61) were collected in screened houses. No significant differences in the densities of the mosquitoes were detected in outdoor collections. Significantly fewer *Anopheles funestus* were collected indoors from houses with painted walls (RR = 0.05, 95% CI 0.01–0.38) while cooking in the house was associated with significantly lower numbers of *Anopheles gambiae* Complex indoors (RR = 0.60, 95% CI 0.45–0.79). Nearly all house owners (99.6%) wanted their houses permanently screened, including 97.7% that indicated a willingness to use their own resources. However, 99.2% required training on house screening. The cost of screening a single house was estimated at KES6,162.38 (US$61.62).

**Conclusion:**

Simple house modification by eave screening has the potential to reduce the indoor occurrence of both *Anopheles* and *Culex* mosquito species. Community acceptance was very high although education and mobilization may be needed for community uptake of house modification for vector control. Intersectoral collaboration and favourable government policies on housing are important links towards the adoption of house improvements for malaria control.

## Background

Substantial progress has been made globally in the fight against malaria in the past two decades [1]. The decline has been attributable to the scale-up of long-lasting insecticidal nets (LLINs) and wide-scale application of indoor residual spraying (IRS) [2]. However, the gains achieved over the past decade and possible future success with these interventions are threatened by widespread insecticide resistance in malaria-endemic areas [3–5], as well as low net coverage and use [6, 7]. Therefore, there is an urgent need for supplementary interventions that are not insecticide-based.

*Anopheles gambiae, Anopheles arabiensis* and *Anopheles funestus*, the primary vectors of malaria in much of sub-Saharan Africa, commonly enter houses and feed on human occupants [8]. Although reports of changing vector behaviour due to sustained LLIN use exist [9–12], consistent evidence of increased exophily and exophagy has not been demonstrated for these malaria vectors. Recent studies in sub-Saharan Africa have reported persistently high estimates of late-night indoor exposure to malaria vectors despite high coverage of insecticide-treated nets [13, 14]. Surveys of *Anopheles* biting behaviour from western Kenya demonstrate late night indoor biting [15, 16] consistent with surveys conducted in the pre-bed net era [17]. These studies indicate that mosquitoes that are strongly adapted to feeding indoors and may not substantially alter their feeding behaviour to seek hosts outdoors due to the presence of treated surfaces indoors, suggesting that other approaches to reducing man-vector contact inside houses may contribute to additional reductions in malaria transmission.

House modification to reduce entry of mosquitoes is a potential tool for the prevention of malaria. Although larval source management and IRS are often credited with the elimination of malaria in Europe and USA, house improvement was likely a major contributor [18]. Observational studies in sub-Saharan Africa suggest a similar potential for improved housing as an approach to reduce malaria transmission [19–21]. Multiple entomological studies in Africa have shown reductions in indoor-resting mosquitoes in houses with closed eaves either by observation of existing house structures [22–24] or through experimental manipulation [20, 25–28], while cluster randomized trials in Ethiopia and The Gambia have demonstrated impacts of house screening on malaria incidence [29] and anaemia [27]. Other approaches to limit mosquito entry into houses, such as eave tubes, have also been shown to reduce malaria incidence [30]. However, a systematic review of data existing at the time concluded that while there was some evidence that house modification reduced transmission and disease due to malaria, the data were limited [31]. Currently, the World Health Organization (WHO) Global Malaria Programme provides a conditional recommendation for house screening based on the low certainty of evidence [32]. The impact of eave screening on indoor occurrence of mosquitoes and community perceptions of eave screening for mosquito control were evaluated to provide evidence for improved housing as a malaria control intervention in western Kenya.

## Methods

### Study site

The research was conducted in Kisian (0° 04′ 16″S 34° 40′ 38″ E), Tiengre (0° 04′ 50″ S 34° 41′ 21″ E) and Rota villages (0° 05′ 36″ S 34° 40′ 28.0″ E) of Kisumu County in western Kenya, near the shores of the Winam Gulf of Lake Victoria between February and September 2013. The landscape is a flat lake plain coursed with streams, and is heavily devoted to cultivation and grazing agriculture, with scattered shrubs and trees amongst human dwellings and various small buildings, dirt roads and footpaths. Most residents are of the Luo ethnic group, subsisting on farming, fishing and trade, and live in small houses clustered into family social units, called compounds [33]. Cattle are often corralled at night within the compounds.

### Houses

**Fig. 1:**
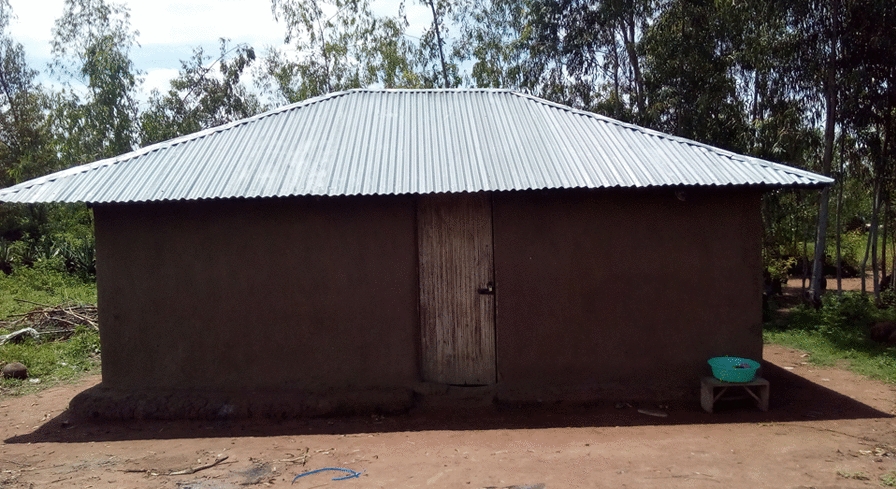
A typical house design commonly found in rural western Kenya

The traditional and modernized Luo house design and construction has been reviewed [33, 34]. Houses are typically rectangular and constructed of stick frames (wattle), compacted soil or cement foundation, and dirt or cement floor. Walls consist of wood ash, mud and cattle dung daubed into the wattle and either left rough (unfinished) or finished with hand smoothing called ‘smearing’ (Fig. 1). Some houses have cement blocks or poured cement walls. Houses are most often roofed with corrugated iron sheets nailed to ceiling joists, but occasionally are roofed with grass thatch or clay tile. Roofs of corrugated iron are usually a simple open gable design, but some houses have hipped roofs. Doors and windows are unframed or framed with wood to create a jam and sash but are often poorly hung. Walls rise to a wall plate topped with wooden pole or rough timber headers. Roof rafters, fastened to these headers with nails, extend upward from the wall plate to one or more tie beams, which are either poles or rough finished timber and are supported by walls. Typically, the roof rafters and roofing material extend as an eave externally hung past the wall dimensions to allow rainwater to drip and drain away from the foundation. A fascia may or may not be present, but the roof rafters at the wall position lack a soffit, leaving the eaves open to the outside air. This open space permits entry and exit of mosquitoes, as do presumably any spaces around windows and door jams.

### Structure of the field study

**Fig. 2:**
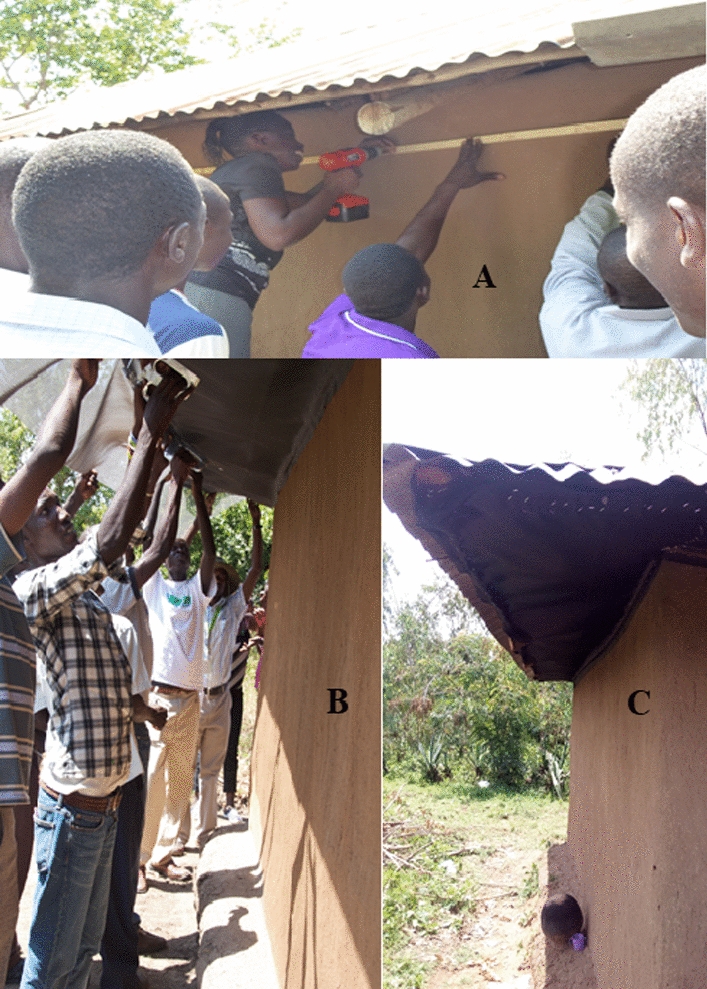
Eave screening: **A** screwing of wood batting on the wall; **B** attaching the PVC insect screen material to the wood batting at the overhanging edge of the roof with a handheld staple gun; and **C** PVC insect screen fixed within the eave space and sewn with thread and needle at the end of successive sheets

A total of 120 pairs of houses were enrolled in February 2013. Neighbouring houses no more than 50 m apart with open eaves and similar roof and wall construction formed a pair. Within each pair, one house was randomly chosen to receive eave screening in March, while the other house in each pair was left unscreened until September 2013, when the eaves of these houses were screened as a benefit to the participants. Screening was accomplished mostly with locally available materials. The screen material was a polyvinyl chloride (PVC) insect mesh. It was cut to shape in long sheets in the field. Wood lathe strips were secured to the upper exterior walls with wood screws (Fig. 2A). One long side of the screen sheet was stapled to the lathe, and the other long side was stapled to the wood pole or rough lumber, forming the eave support (Fig. 2B). Staples were 1/4″ or 3/8″ size and were applied with handheld staple guns (Arrow, Detroit, MI, USA). Eave screen material was sewn with thread and needle at the ends as successive sheets were hung to the eaves as described above (Fig. 2C). Openings in the screen eaves occasionally occurred because of undulations from mud produced during the wall construction. They were filled by folding the screen material into place and securing it with staples onto shims made from the wooden lathes. If doors were poorly hung leaving gaps between the door and door jam, the gaps were fitted with screens as well, but windows were not screened.

### Mosquito sampling

Mosquitoes were sampled monthly indoors and outdoors with battery-powered aspirators [35] between April and July 2013. A crew of two sampled each house. One used the aspirator indoors to retrieve mosquitoes from underneath and behind furniture, resting on walls and in other locations in the house. The indoor collector spent 10 min in each house. He passed sample containers to the second person working outside the house, who removed mosquitoes from the sample containers, transferred them to 30 × 30 × 30 cm mosquito cages, and then aspirated them using mouth aspirators to holding cups provisioned with moistened cotton balls. After completing the indoor sample, the field worker used the battery powered aspirator to sample resting mosquitoes from two clay pots [36] set within 5 m of each house with the mouth of each pot directed to the wall. Samples were taken at monthly intervals in April, May, June, and July.

*Anopheles* mosquitoes were examined morphologically to identify species. Based on the morphological identification results, the samples were subjected to polymerase chain reaction (PCR) to identify sibling species of the *Anopheles gambiae* complex [37] and *Anopheles funestus* group [38].

### Questionnaires

A structured questionnaire was administered to an adult member of the household, usually the primary female head of household who had offered the original consent. The same questions were asked four times after every 2 months, once before screening in February, and three times after screening of the houses, in April, June and August 2013. The questions encompassed the following themes: (1) Attitude towards eave screening; (2) use of bed nets; and (3) use of anti-mosquito methods such as mosquito coils. A survey of the screening material was made approximately 8 years after installation to assess how many screens remained intact.

### Data management and analysis

Field entomological data collection used Visual CE software run on personal digital assistants (PDA). Data entry screens used drop-down menus and automatic data checks to reduce errors. A unique collection code was generated by the PDA for every house sampled. Additionally, a house code, which was a combination of the first three letters of the district name, village name, name of the compound head, and a number representing collection in each compound was also used to identify each collection uniquely. Individual mosquitoes from each collection were placed in Eppendorf tubes labelled with pre-printed barcodes and linked to the field data by house code and collection code. Results of species identification by PCR were linked to individual mosquitoes by the unique barcode label.

Data analysis was performed using R statistical software version 4.1.2. The risk ratio (RR) was used to assess the statistical significance of differences in mosquito densities between screened and unscreened houses. Data were fitted using Generalized Linear Mixed Effects Statistical Models (GLMMs). Since the data were over-dispersed, the package GLMM using Template Model Builder (glmmTMB) was used to fit negative binomial distribution models for the analysis of mosquito numbers. The numbers of female *Anopheles* and *Culex* species were assessed as a function intervention status (screened or unscreened) as a fixed effect, while paired houses and sampling period were treated as a random effect. To obtain the RR and confidence intervals, the model coefficients were exponentiated. The modelled per cent reduction in mosquito densities in screened houses compared to unscreened houses was calculated as 100*(1-RR). Models were adjusted for wall type, windows intact (windows with or without obvious gaps in and/or around them), net use, and cooking in the house.

### Ethical considerations

The study was approved by the Kenya Medical Research Institute/ Scientific and Ethics Review Unit (KEMRI/SERU, protocol number 2269) and by the US Centers for Disease Control and Prevention (CDC) through a reliance agreement with KEMRI/SERU (CDC IRB 6401), and by the Michigan State University Institutional Review Board (MSU IRB 12-611). A written consent was obtained from household heads for eave screening, collection of mosquitoes, and administration of the questionnaire.

## Results

### Mosquitos catch sizes

Of the original 240 households enrolled in the study, five withdrew and at times others were unavailable for sampling if the householders were absent, the door locked, or the outdoor collection pots moved. A total of 909 indoor and 895 outdoor collections were performed over the four-month period against the expected 960 efforts per trapping location. Overall, 2412 female and 2158 male *Anopheles*, and 14,689 female and 18,746 male *Culex* species were collected. Of the *Anopheles gambiae* Complex 1017 were analysed for species identification by PCR and 844 (83%) were identified to be *Anopheles arabiensis* while 173 (17%) were *Anopheles gambiae s.s.* Of the *Anopheles funestus* Group, 936 (90%) were identified to be *Anopheles funestus s.s.,* the remainder were not identified.

**Fig. 3:**
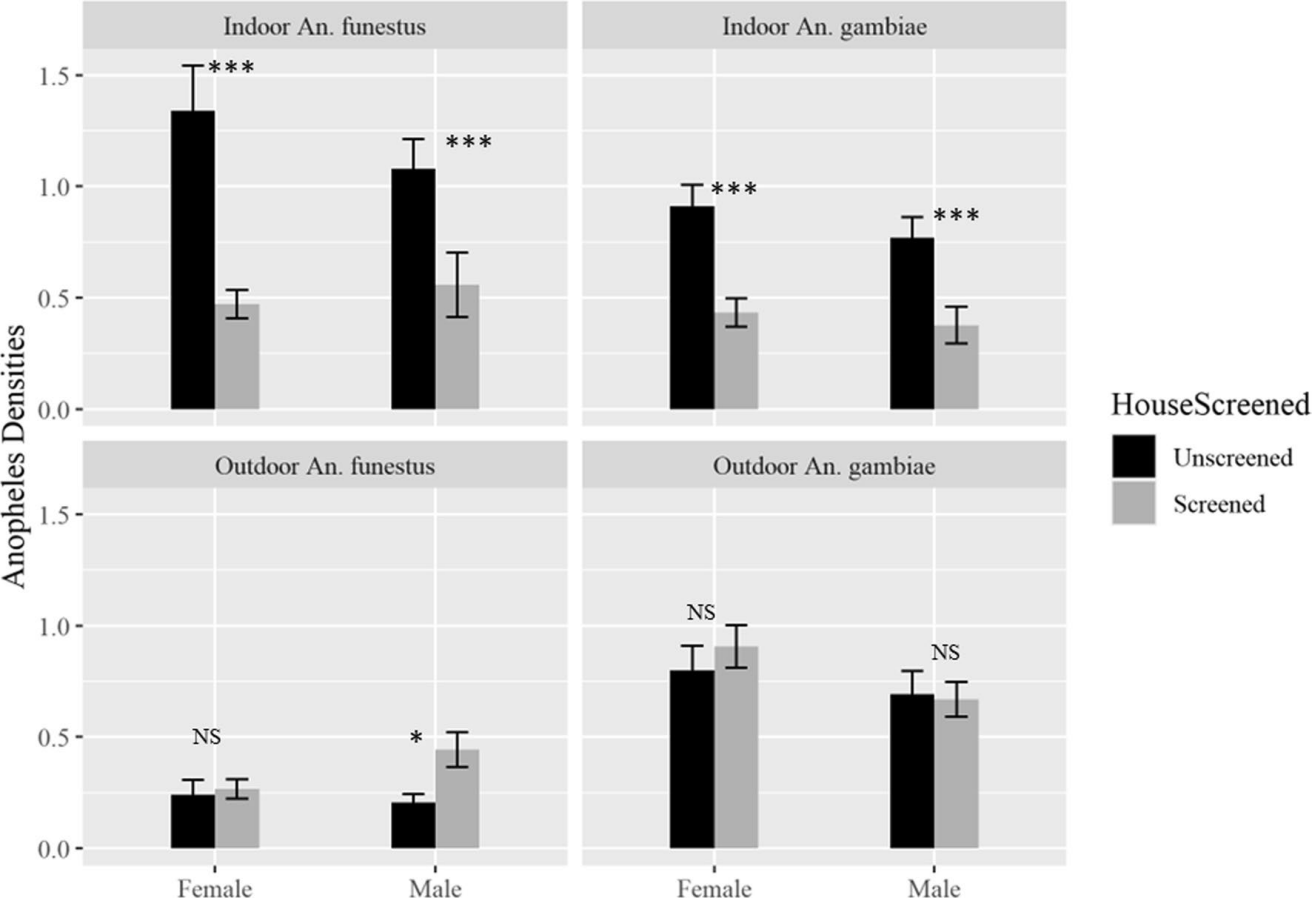
Comparison of mean number of male and female *Anopheles funestus* and *Anopheles gambiae* Complex densities between screened and unscreened houses indoors and outdoors. The asterisks show level of significance between the screened and unscreened houses, ***P < 0.001, *P = 0.05 and NS: Not significant

**Fig. 4:**
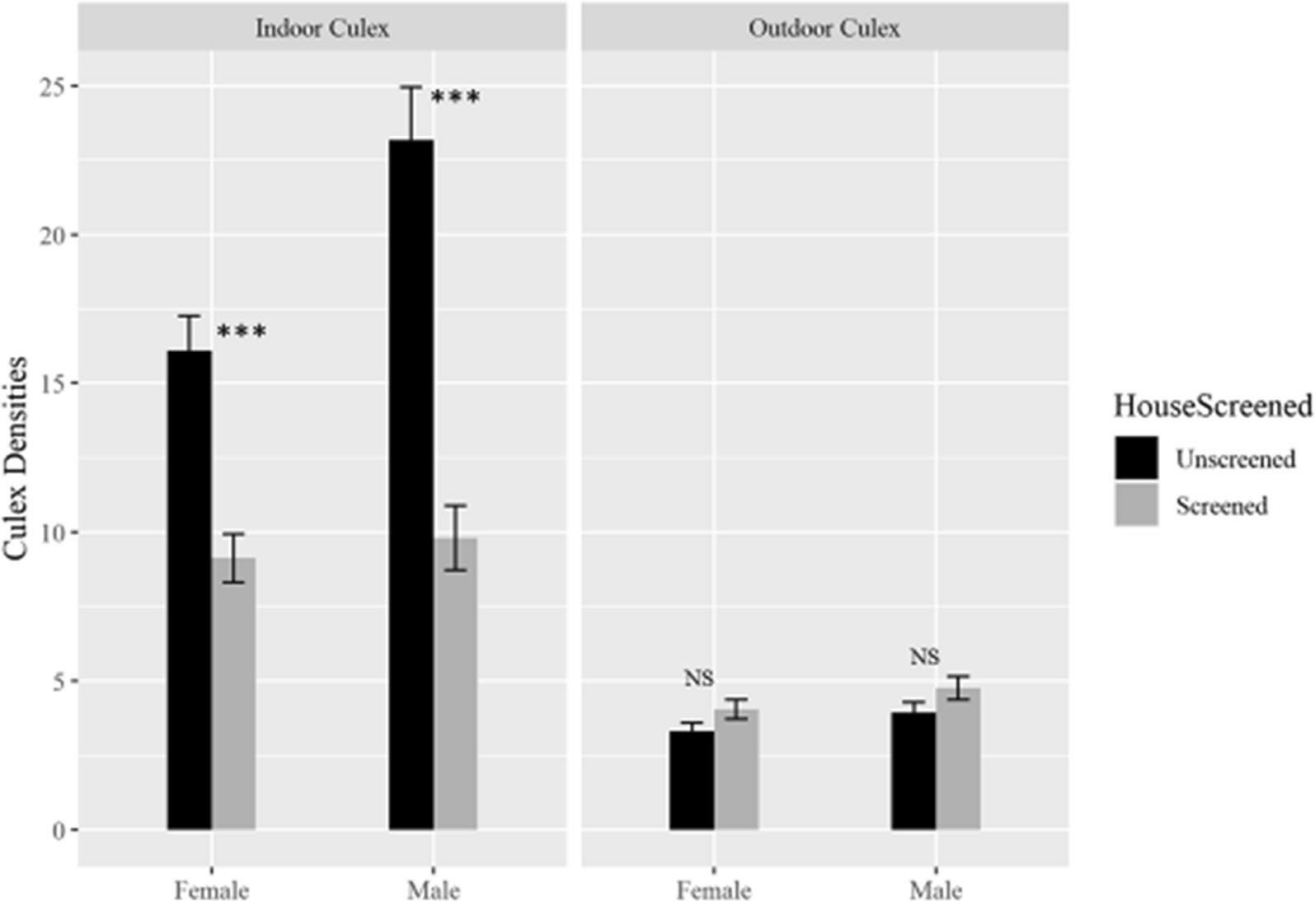
Comparison of mean number of male and female *Culex* species densities between screened and unscreened houses indoors and outdoors. The asterisks show level of significance between the screened and unscreened houses, ***P < 0.001 and NS = Not significant

The mean number of each mosquito species collected indoors and outdoors from screened and unscreened houses is shown in Table 1, and Figs. 3 and 4. The mean number of female *Anopheles funestus*, *Anopheles gambiae* and *Culex* species caught inside screened houses was significantly lower compared to unscreened houses. Based on the modelled estimates, there were 60% fewer *Anopheles funestus* (RR = 0.40, 95% CI 0.29–0.55, p < 0.001), 54% fewer *Anopheles gambiae* complex (RR = 0.46, 95% CI 0.41–0.62, p < 0.001) and 47% fewer *Culex* species (RR = 0.53, 95% CI 0.45–0.61, p < 0.001) in screened houses compared to unscreened houses. Similarly, there were 63% fewer male *Anopheles funestus* (RR = 0.37, 95% CI 0.26–0.53, p < 0.001), 60% fewer male *Anopheles gambiae* complex (RR = 0.40, 95% CI 0.28–0.57, p < 0.001) and 64% fewer male *Culex* species (RR = 0.36, 95% CI 0.30–0.43, P < 0.001) in screened houses compared to unscreened houses. From outdoor collections, there were no significant differences in the number of female and male *Anopheles funestus*, *Anopheles gambiae* Complex or *Culex* species, except for male *Anopheles funestus* where significantly higher numbers were observed around screened houses compared to unscreened ones, (RR = 1.59, 95% CI 1.00–2.53, p = 0.05) (Table 1).

**Table 1:** Comparison of mean number of female and male *Anopheles funestus*, *Anopheles gambiae* Complex and *Culex* species collected indoors and outdoors by aspiration between screened and unscreened houses

Collection location	Category	Mosquito species	Intervention status	Mosquito numbers	Mean (95% CI)	RR (95% CI)	P-value
Indoors	Female	*Anopheles funestus*	Screened	219	0.47 (0.34–0.60)	0.40 (0.29–0.55)	**< 0.001**
			Unscreened	593	1.34 (0.94–1.74)	Ref	
		*Anopheles gambiae*	Screened	202	0.43 (0.31–0.56)	0.46 (0.34–0.62)	**< 0.001**
			Unscreened	403	0.91 (0.72–1.10)	Ref	
		*Culex* species	Screened	4256	9.13 (7.55–10.72)	0.53 (0.45–0.61)	**< 0.001**
			Unscreened	7128	16.09 (13.80–18.38)	Ref	
	Male	*Anopheles funestus*	Screened	260	0.56 (0.28–0.84)	0.37 (0.26–0.53)	**< 0.001**
			Unscreened	478	1.08 (0.82–1.34)	Ref	
		*Anopheles gambiae*	Screened	175	0.38 (0.21–0.54)	0.40 (0.28–0.57)	**< 0.001**
			Unscreened	341	0.77 (0.59–0.95)	Ref	
		*Culex* species	Screened	4573	9.81 (7.67–11.95)	0.36 (0.30–0.43)	**< 0.001**
			Unscreened	10,265	23.17 (19.70–26.65)	Ref	
Outdoors	Female	*Anopheles funestus*	Screened	123	0.27 (0.18–0.35)	1.08 (0.72–1.63)	0.70
			Unscreened	105	0.24 (0.12–0.37)	Ref	
		*Anopheles gambiae*	Screened	418	0.91 (0.72–1.09)	1.23 (0.94–1.62)	0.13
			Unscreened	347	0.80 (0.58–1.02)	Ref	
		*Culex* species	Screened	1867	4.05 (3.40–4.69)	1.11 (0.90–1.37)	0.33
			Unscreened	1438	3.31 (2.77–3.86)	Ref	
	Male	*Anopheles funestus*	Screened	204	0.44 (0.29–0.60)	1.59 (1.00–2.53)	**0.05**
			Unscreened	90	0.21 (0.14–0.28)	Ref	
		*Anopheles gambiae*	Screened	308	0.67 (0.51–0.82)	1.09 (0.79–1.51)	0.58
			Unscreened	301	0.69 (0.49–0.89)	Ref	
		*Culex* species	Screened	2196	4.76 (4.00–5.53)	1.10 (0.87–1.39)	0.42
			Unscreened	1712	3.94 (3.26–4.63)	Ref

### Other factors affecting female mosquito numbers

Table 2 presents modelled estimates of the effect of wall type, window condition (whether intact or not), use of nets and cooking in the houses on the indoor occurrence of female *Anopheles funestus, Anopheles gambiae* and *Culex* species in screened and unscreened houses as measured by indoor aspiration. Significantly fewer *Anopheles funestus* were sampled from houses with painted cement walls compared to houses with unfinished brick-walled houses (RR = 0.05, 95% CI 0.01–0.38, p = 0.04). Houses that had intact windows without obvious gaps on/around the window had significantly lower numbers of female *Anopheles funestus* compared to houses that had intact windows (RR = 0.66, 95% CI 0.44–0.98, p = 0.04). Significantly lower numbers of female *Anopheles funestus* were observed in houses where at least a single household member slept under a bed net the night before collection compared to houses where no one used a bed net (RR = 0.45, 95% CI 0.29–0.70, p = 0.0003). For female *Anopheles gambiae*, lighting a fire for cooking indoors the night before collection was associated with significantly lower numbers of this species indoors (RR = 0.60, 95% CI 0.45–0.79, p = 0.0002). For female *Culex* species, intact windows and net use by at least a single household member were associated with significantly lower numbers indoors (RR = 0.65, 95% CI 0.54–0.80, p < 0.001) and (RR = 0.79, 95% CI 0.62–1.00, p = 0.05).

**Table 2:** Comparison of mean number of female *Anopheles funestus*, *Anopheles gambia*e and *Culex* species collected indoors by wall type, window condition, net use, and cook in the house

Mosquito species	Parameter	Level	Mean	Risk ratio	Lower CL	Upper CL	P-value
*Anopheles funestus*	Wall type	Cement	0.69	0.27	0.05	1.58	0.15
		Mud	0.88	0.31	0.04	2.32	0.25
		Painted cement	0.13	0.05	0.01	0.38	**0.004**
		Plastered mud	0.64	0.26	0.05	1.34	0.11
		Brick	4.50	Ref			
	Windows intact	Yes	0.62	0.66	0.44	0.98	**0.04**
		No	0.78	Ref			
	Net use	Yes	0.45	0.45	0.29	0.70	**0.0003**
		No	1.56	Ref			
	Cook in the house	Yes	0.59	0.92	0.65	1.31	0.66
		No	0.71	Ref			
*Anopheles gambiae*	Wall type	Cement	0.49	1.15	0.23	5.62	0.86
		Mud	0.54	1.28	0.21	7.70	0.79
		Painted cement	0.32	0.70	0.13	3.75	0.68
		Plastered mud	0.61	1.31	0.29	5.87	0.72
		Brick	0.40	Ref			
	Windows intact	Yes	0.55	0.75	0.57	1.02	0.07
		No	0.74	Ref			
	Net use	Yes	0.54	0.76	0.55	1.07	0.12
		No	0.81	Ref			
	Cook in the house	Yes	0.42	0.60	0.45	0.79	**0.0002**
		No	0.74	Ref			
*Culex* species	Wall type	Cement	9.86	2.48	0.84	7.27	0.10
		Mud	5.63	2.46	0.73	8.25	0.15
		Painted cement	6.30	2.51	0.82	7.66	0.12
		Plastered mud	10.86	3.04	1.10	8.42	0.03
		Brick	2.20	Ref			
	Windows intact	Yes	9.62	0.65	0.54	0.80	**< 0.001**
		No	13.47	Ref			
	Net use	Yes	9.87	0.79	0.62	1.00	**0.05**
		No	13.08	Ref			
	Cook in the house	Yes	9.96	0.89	0.74	1.08	0.25
		No	10.93	Ref			

### Response to questionnaire

Prevention of mosquito entry was repeatedly cited as the major advantage of eave screening by households that had screened eaves, 94% (111/117), 91% (106/117) and 99% (114/115) in rounds two, three and four of questionnaire, respectively. Other advantages of eave screening identified included prevention of entry of animals such as cats, rats, snakes, bats, and flying insects as well as making the house warmer, preventing malaria and making the house more attractive esthetically. From four rounds of questionnaires, nearly all households 99.6% (926/930) wanted their houses screened permanently, 99.2% (922/929) indicated that they would require training on how to screen their own houses, and 94.6% indicated a willingness to use their own resources to screen their own houses. However, one disadvantage of eave screening identified by the residents was that it prevented exiting of mosquitoes that had entered the house.

### Other vector control methods

Most of the households interviewed (97.1%, 903/930) had LLINs, while 0.3% (3/930) used mosquito coils. Only 1.9% (18/930) used no vector control method in addition to screening. Most (98.6%, 890/903) of those who reported having bed nets had them hanging over their sleeping areas while 1.4% (13/903) of those who reported owning bed nets but did not hang them in their sleeping areas. Of all the households interviewed over the study period, 15.9% (148/930) reported that some members of the household did not sleep under a bed net in the night before the interview. The main reason identified for not sleeping under a bed net was lack of enough nets for all members of the households (82.4%, 122/148). Other reasons for not using a bed net included absence of mosquitoes (1.9%, 18/930), nets were hard to hang (2.0%, 19/930), or the house was too warm (6.0%, 56/930).

### Cost of eave screening

The direct cost required to screen the eaves of a single standard house was estimated at KES5775 (US$57.75). The total equipment cost was KES46,485.6 (US$464.40), which covered all installations. The cost of equipment per house was conservatively estimated by dividing the total equipment cost by the number of houses installed with eave screens (120) for a per house cost of KES387.38 (US$3.87). Therefore, the cost of screening a single house was estimated at KES6162.38 (US$61.62) as of 2013 when the screening was conducted (Table 3).

**Table 3:** Estimates of costs of screening the eaves of a single standard house (in the 2023)

Cost category	Item	Quantity per house	Unit cost	Total cost (KES)	Total cost (USD)
Direct cost per house (A)	PVC insect screen	0.5 Rolls	2000	2000	20
	Wood (lathe)	110 feet	15	1650	16.5
	Screws	140 pieces	10	1400	14
	Staple pins	1 packet	225	225	2.25
	Labor costs	1 person-day per house*	500	500	5
Subtotal (Direct cost per house (A))				5775	57.75
Cost for equipment per team that screened 120 houses (B)	Transportation of timber	Single trip	5000	5000	50
	Drill bits	3 pieces	7500	7500	75
	Screwdriver	1 piece	300	300	3
	Wood saw	1 piece	300	300	3
	Tape measure	1 piece	1500	1500	15
	Hammer	1 piece	700	700	7
	Power Drill	1 piece	19,000	19,000	190
	Staple guns	3 pieces	4062	12,186	121.86
Subtotal (Aggregate cost)				46,486	464.86
Cost of equipment per house (Equipment aggregate cost/120), (B)				387.38	3.87
Approximate cost per house (A + B)				6162.38	61.62

*Labourers who install the screens worked in teams of three. Each team was able to complete on average three houses per day

### Current status of the previously screened houses

**Fig. 5:**
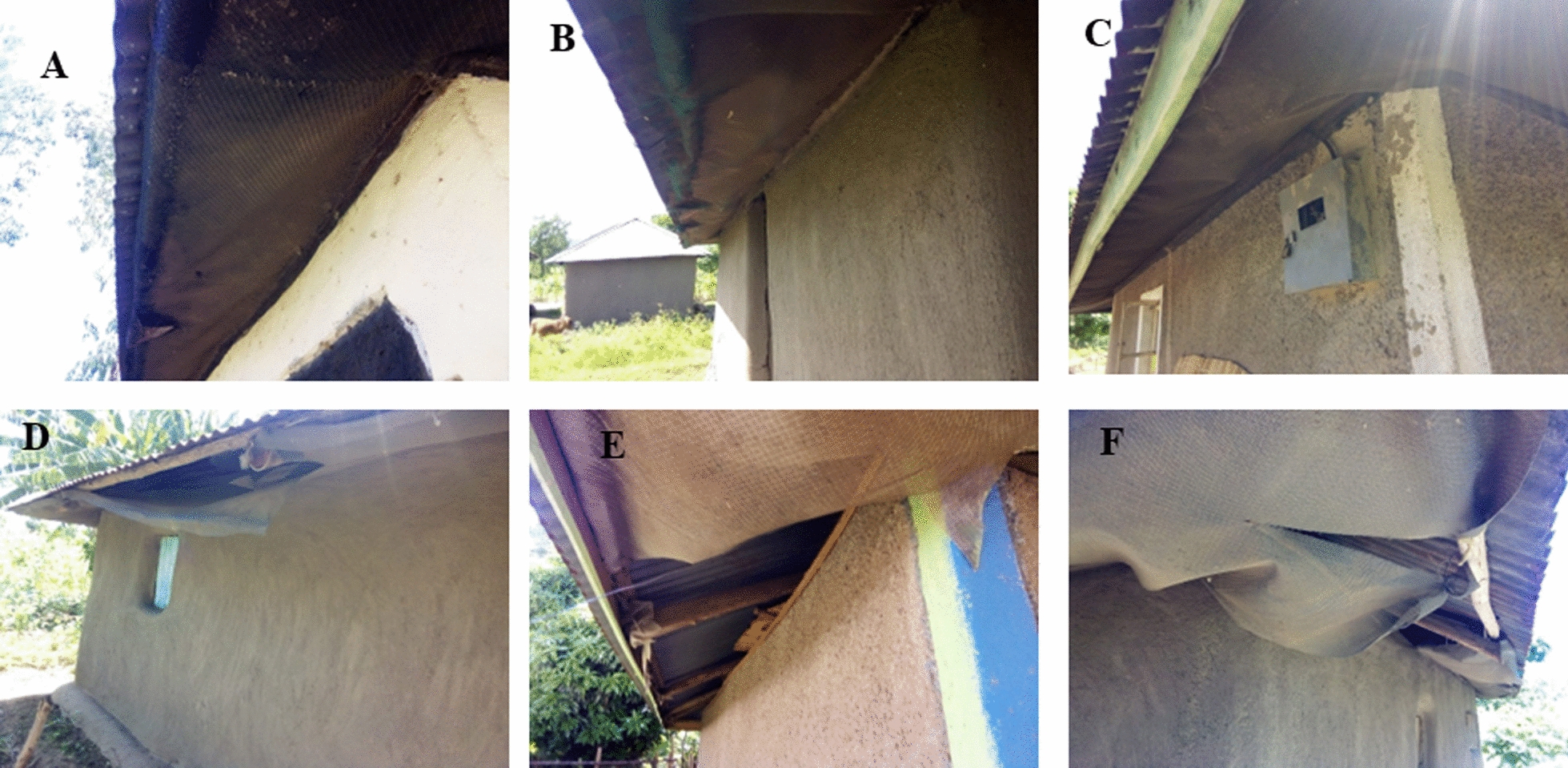
Houses with intact screen (**A**–**C**) and those with damaged screens (**D**–**F**), 8 years post screening

A survey was conducted to check on the status of screening of houses used in the current study, approximately 8 years after screening. Out of the 235 houses that participated, information was obtained from 234 structures. Nearly half of the houses (104; 44.4%) had been demolished at the time of the survey and a similar number (104, 44.4%) no longer had screens. Eleven (4.7%) of the houses had intact screens in good condition (Fig. 5A–C) while 15 (6.4%) had damaged screens (Fig. 5D–F).

## Discussion

The results demonstrate a significant reduction in indoor-resting densities of *Anopheles funestus, Anopheles gambiae* Complex and *Culex* species attributed to eave screening. Other factors associated with reduced mosquito numbers inside houses included wall type, the presence of intact windows, cooking in the house, and the usage of LLINs. However, the association between these other factors and reduced mosquito numbers varied with mosquito species. There were no differences in the numbers of mosquitoes collected outdoors in the peri-domestic spaces of screened and unscreened houses. In interviews with participating households, most community members indicated a desire and willingness to install eave screens but reported a need to receive training in how to implement this potential mosquito control intervention.

Eaves have been demonstrated to be the main entry route for *Anopheles* mosquitoes into houses [39]. Most houses in rural Africa have open eaves, which are important for ventilation to allow natural light and fresh air into the houses. At night when doors and windows are closed, eave spaces become the primary route through which air flows into and out of the house. As air laden with odours from occupants indoors flows out through eave spaces, mosquitoes fly upwind [40] following the odour source. They are then funneled indoors by the roof hanging over the eave space [39]. Reductions in indoor *Anopheles* densities due to house modification have been demonstrated previously [28, 41–43]. Consistent with the previous studies, the potential of eave screening in reducing numbers of mosquitoes indoors and the risk of malaria transmission is demonstrated.

While eaves are the primary route of entry into houses, mosquitoes may still gain entry through doors and windows. In the current study, houses that had intact windows, with no obvious gaps around the frame or shutter, had a significantly reduced risk of *Anopheles funestus* and *Culex* species indoors. Numbers of *Anopheles gambiae* Complex were lower in houses with intact windows although the difference was not statistically significant. Increased presence of *Anopheles funestus* in houses that had gaps around the windows indicates this species may enter the house through routes other than the eaves. *Anopheles funestus* are strongly endophilic and endophagic [44–47] and are more inclined to indoor entry. The species is therefore more likely to be impacted by improved housing conditions, as observe in this study. *Anopheles arabiensis* was the most dominant species (83%) of the *Anopheles gambiae* Complex population and are more associated with outdoor feeding and resting [44–47]. The species is also reportedly less impacted by indoor interventions [16, 46]. This possibly explains why other house factors such as presence of intact windows, wall type and net usage did not have a significant reduction in the number of the species indoors. Screening doors and windows and closing openings on eaves and walls with mud was observed to reduce the overall indoor densities of *Anopheles arabiensis* by 40% in southwest Ethiopia [26]. In a Gambian village, different prototypes of doors reduced the number of house-entering mosquitoes by 59–77% in comparison with the control houses [48]. However, the use of screened doors for mosquito control is faced with challenges that may compromise their efficacy. Doors were frequently found to be propped open with sticks or a heavy object to allow easy access into the home and to promote cross ventilation [49], while in grass-thatched houses, doors were observed to be incompatible since low-hanging roofs, preventing the opening of screened doors outwards [26]. Consequently, further studies are necessary to underscore the additional benefits of screened doors and windows and to develop suitable designs adaptable to different house structures. Community engagement to discourage household residents from leaving the doors open for extended periods may also be required to ensure their efficacy.

Improved housing conditions in rural Africa are largely associated with plastered, painted walls with iron roofs and closed eaves. Compared to traditional housing designs with thatched roofs and mud walls, modern house designs are commonly associated with lower numbers of mosquitoes. In addition to screening, houses with painted walls were observed to have reduced risk of *Anopheles funestus* indoors. Painted walls are indicative of modern housing conditions and have been associated with reduced clinical malaria when compared to traditional housing [19]. Painting of walls is mostly performed with white or light colours that tend to brighten the house, and which may deter indoor resting of mosquitoes. Previous studies with *Aedes* species demonstrated that mosquitoes are attracted to red and black clothes, with darker colours being more attractive than lighter ones [50]. Although not tested in the current study, metal-roofed houses have also been associated with reduced mosquito survival [51]. Although blocking access points through which mosquitoes enter the houses likely has the greatest potential for reducing malaria transmission, other incremental house improvements may also affect mosquito behaviour and survival and contribute to reduced malaria transmission.

Eave screening of houses prevents mosquitoes from entering houses. It is not clear how strongly endophilic and endophagic malaria vectors such as *Anopheles funestus* and *Anopheles gambiae s.s.* will respond and whether they will simply shift to feeding and resting outdoors. The results indicated a non-significant increase in the number of mosquitoes collected outdoors. Although not statistically significant, it is possible that the differences would be greater if the houses were fully screened at a larger coverage as mosquitoes in this study may have simply been diverted to neighbouring, unscreened houses. However, it is also possible that high coverage of improved housing may have a community effect as has been observed with insecticide treated nets (ITNs) [52]. With high coverage, anthropophilic, endophilic mosquitoes may have limited opportunities to feed on their preferred host and may experience substantial population reductions at high coverage. Studies on the biting time and location of *Anopheles funestus* indicate that it largely feeds indoors late at night despite the scale-up of ITNs [13, 15, 16]. These observations suggest that this species may be constrained in its ability to adapt to feeding and resting outdoors. Larger scale studies are required to assess the impact of implementing house modification at high coverage on mosquito populations as well as their behavioural response to the intervention.

The study community in rural western Kenya demonstrated a willingness of to receive training on house screening, spend their own resources to improve their houses and have them permanently screened. This expression of willingness was likely prompted by communication from the study team and the observed immediate benefits of house screening by the homeowners. However, over the years, with no contact or support from the study team, nearly half of the households were demolished and, of those houses that remained, most had removed the screens. Of the houses where screens remained, more than half were damaged. Household owners were not asked about the causes of damage to the screens over the years. However, based on the observations during the initial 4-month study, it is suspected that the material used could develop small holes, and once a hole developed, it would easily propagate across the fabric. Better quality insect screen materials, such as fibreglass, would likely be more durable although more costly. Durable screening material would likely provide protection for the lifetime of the house and, with costs spread across many years, would possibly be a cost-effective approach to malaria prevention and control.

House screening recently received a World Health Organization (WHO) conditional recommendation [32] as a malaria prevention tool but additional evidence on malaria transmission reduction is required to attain a full recommendation. Should house modification be recommended as a malaria prevention tool, innovative approaches to financing will be required. The cost of eave screening was approximated at US$61.62 per house. Other vector control tools are costed at average US$2.00 for LLINs [53] and US$8.26 for IRS with pirimiphos-methyl [54] per person protected. Comparatively, house modification is costly in the initial application. However, it is cost effective over time because a single installation would possibly last the entire period of the structure modified. Donor-led funding of house modification in sub-Saharan Africa may be limited due to the wide variety of house structures that will likely preclude a one size fits all approach as is possible with ITNs or IRS. Ultimately, improved housing will likely need to be financed largely by individual homeowners and costs factored into the overall house construction cost. Africa is one the fastest growing economies in the world with a 6% annual increase in the gross domestic product expected by 2025 [55]. Associated increased personal wealth has led to housing improvements, such as replacement of traditional thatch with metal or tile roofs [41]. However, the large initial investment in improved houses is likely a substantial barrier for many poor families in rural Africa. Strong donor involvement could contribute to the uptake of improved housing through social and behavioural change interventions. This would be targeted at homeowners, facilitation of intersectoral engagement to spur innovation in house improvement and advocacy for policy development, such as regulations for new construction and/or tax policies that promote improved housing.

Like other studies, this evaluation had limitations that merit consideration. Screening was only conducted on the eaves while doors and windows remained unscreened. The doors and windows provided potential entry routes for mosquitoes resulting in a continued risk of malaria transmission. Full screening of eaves, doors and windows may have provided even greater impact on mosquito numbers indoors although this approach would have incurred substantial additional costs. Entomological monitoring of the impact of eave screening was conducted for only 4 months due to limited funding. The period of monitoring covered the high transmission season when the greatest impact would be expected and extending the study across an entire year may have resulted in lower estimated impacts on mosquito numbers. The follow-up survey of the presence and physical integrity of the screen material eight years after screening did not include interviews with the community to underpin reasons for removal and failure to replace the screen material. However, this study suggests a substantial impact on malaria transmission could be achieved through house screening when the risk of malaria is highest. Additionally, this report provides information on three major points of consideration under the WHO’s conditional recommendation: namely, delivery and maintenance of screening, structural condition of the residential houses to allow installation of screen and feasibility of resources needed for implementation [32].

## Conclusion

Simple house modification by screening of eaves significantly reduced numbers of malaria vectors indoors as well as nuisance mosquitoes. With sustained indoor biting and resting of malaria-transmitting mosquitoes, house modification holds great potential for malaria control and elimination. Simple incremental structural modifications of existing houses and incorporation of screening in new houses for malaria control in Africa will require effective stakeholder engagement, advocacy and relevant policy development.

## Data Availability

Data available at request from the corresponding author.

## References

[1] WHO (2021). World Malaria Report 2021.

[2] Bhatt S, Weiss DJ, Cameron E, Bisanzio D, Mappin B, Dalrymple U (2015). The effect of malaria control on *Plasmodium falciparum* in Africa between 2000 and 2015. Nature.

[3] Ochomo EO, Bayoh NM, Walker ED, Abongo BO, Ombok MO, Ouma C (2013). The efficacy of long-lasting nets with declining physical integrity may be compromised in areas with high levels of pyrethroid resistance. Malar J.

[4] Edi CV, Koudou BG, Jones CM, Weetman D, Ranson H (2012). Multiple-insecticide resistance in *Anopheles gambiae* mosquitoes, Southern Cote d'Ivoire. Emerg Infect Dis.

[5] Sougoufara S, Doucoure S, Backe Sembene PM, Harry M, Sokhna C (2017). Challenges for malaria vector control in sub-Saharan Africa: resistance and behavioral adaptations in *Anopheles* populations. J Vector Borne Dis.

[6] Solomon T, Loha E, Deressa W, Gari T, Lindtjørn B (2018). Low use of long-lasting insecticidal nets for malaria prevention in south-central Ethiopia: a community-based cohort study. PLoS ONE.

[7] Yang GG, Kim D, Pham A, Paul CJ (2018). A meta-regression analysis of the effectiveness of mosquito nets for malaria control: the value of long-lasting insecticide nets. Int J Environ Res Public Health.

[8] Seyoum A, Sikaala CH, Chanda J, Chinula D, Ntamatungiro AJ, Hawela M (2012). Human exposure to anopheline mosquitoes occurs primarily indoors, even for users of insecticide-treated nets in Luangwa Valley, South-east Zambia. Parasit Vectors.

[9] Russell TL, Govella NJ, Azizi S, Drakeley CJ, Kachur SP, Killeen GF (2011). Increased proportions of outdoor feeding among residual malaria vector populations following increased use of insecticide-treated nets in rural Tanzania. Malar J.

[10] Meyers JI, Pathikonda S, Popkin-Hall ZR, Medeiros MC, Fuseini G, Matias A (2016). Increasing outdoor host-seeking in *Anopheles gambiae* over 6 years of vector control on Bioko Island. Malar J.

[11] Degefa T, Yewhalaw D, Zhou G, Lee MC, Atieli H, Githeko AK (2017). Indoor and outdoor malaria vector surveillance in western Kenya: implications for better understanding of residual transmission. Malar J.

[12] Ototo EN, Mbugi JP, Wanjala CL, Zhou G, Githeko AK, Yan G (2015). Surveillance of malaria vector population density and biting behaviour in western Kenya. Malar J.

[13] Bayoh MN, Walker ED, Kosgei J, Ombok M, Olang GB, Githeko AK (2014). Persistently high estimates of late night, indoor exposure to malaria vectors despite high coverage of insecticide treated nets. Parasit Vectors.

[14] Huho B, Briet O, Seyoum A, Sikaala C, Bayoh N, Gimnig J (2013). Consistently high estimates for the proportion of human exposure to malaria vector populations occurring indoors in rural Africa. Int J Epidemiol.

[15] Abong'o B, Gimnig JE, Longman B, Odongo T, Wekesa C, Webwile A (2021). Comparison of four outdoor mosquito trapping methods as potential replacements for human landing catches in western Kenya. Parasit Vectors.

[16] Abong'o B, Gimnig JE, Torr SJ, Longman B, Omoke D, Muchoki M (2020). Impact of indoor residual spraying with pirimiphos-methyl (Actellic 300CS) on entomological indicators of transmission and malaria case burden in Migori County, western Kenya. Sci Rep.

[17] Chandler JA, Highton RB, Hill MN (1975). Mosquitoes of the Kano Plain, Kenya. I. Results of indoor collections in irrigated and nonirrigated areas using human bait and light traps. J Med Entomol.

[18] Lindsay SW, Emerson PM, Charlwood JD (2002). Reducing malaria by mosquito-proofing houses. Trends Parasitol.

[19] Tusting LS, Ippolito MM, Willey BA, Kleinschmidt I, Dorsey G, Gosling RD (2015). The evidence for improving housing to reduce malaria: a systematic review and meta-analysis. Malar J.

[20] Tusting LS, Bottomley C, Gibson H, Kleinschmidt I, Tatem AJ, Lindsay SW (2017). Housing improvements and malaria risk in sub-Saharan Africa: a multi-country analysis of survey data. PLoS Med.

[21] Rek JC, Alegana V, Arinaitwe E, Cameron E, Kamya MR, Katureebe A (2018). Rapid improvements to rural Ugandan housing and their association with malaria from intense to reduced transmission: a cohort study. Lancet Planet Health.

[22] Animut A, Balkew M, Lindtjørn B (2013). Impact of housing condition on indoor-biting and indoor-resting *Anopheles arabiensis* density in a highland area, central Ethiopia. Malar J.

[23] Wanzirah H, Tusting LS, Arinaitwe E, Katureebe A, Maxwell K, Rek J (2015). Mind the gap: house structure and the risk of malaria in Uganda. PLoS ONE.

[24] Lwetoijera DW, Kiware SS, Mageni ZD, Dongus S, Harris C, Devine GJ (2013). A need for better housing to further reduce indoor malaria transmission in areas with high bed net coverage. Parasit Vectors.

[25] Snetselaar J, Njiru BN, Gachie B, Owigo P, Andriessen R, Glunt K (2017). Eave tubes for malaria control in Africa: prototyping and evaluation against *Anopheles gambiae s.s.* and *Anopheles arabiensis* under semi-field conditions in western Kenya. Malar J.

[26] Massebo F, Lindtjørn B (2013). The effect of screening doors and windows on indoor density of *Anopheles arabiensis* in south-west Ethiopia: a randomized trial. Malar J.

[27] Kirby MJ, Ameh D, Bottomley C, Green C, Jawara M, Milligan PJ (2009). Effect of two different house screening interventions on exposure to malaria vectors and on anaemia in children in The Gambia: a randomised controlled trial. Lancet.

[28] Atieli H, Menya D, Githeko A, Scott T (2009). House design modifications reduce indoor resting malaria vector densities in rice irrigation scheme area in western Kenya. Malar J.

[29] Getawen SK, Ashine T, Massebo F, Woldeyes D, Lindtjorn B (2018). Exploring the impact of house screening intervention on entomological indices and incidence of malaria in Arba Minch town, southwest Ethiopia: a randomized control trial. Acta Trop.

[30] Sternberg ED, Cook J, Alou LPA, Assi SB, Koffi AA, Doudou DT (2021). Impact and cost-effectiveness of a lethal house lure against malaria transmission in central Cote d'Ivoire: a two-arm, cluster-randomised controlled trial. Lancet.

[31] Furnival-Adams J, Olanga EA, Napier M, Garner P (2021). House modifications for preventing malaria. Cochrane Database Syst Rev.

[32] WHO (2022). Guidelines for malaria.

[33] Abonyo DA. Cultural aspects of housing: a case of the Luo in Kisumu Town, Kenya. 33rd IAHS World Congress on Housing, Pretoria, South Africa; 2005.

[34] Knols BG, Njiru BN, Mathenge EM, Mukabana WR, Beier JC, Killeen GF (2002). MalariaSphere: a greenhouse-enclosed simulation of a natural *Anopheles gambiae* (Diptera: Culicidae) ecosystem in western Kenya. Malar J.

[35] Vazquez-Prokopec GM, Galvin WA, Kelly R, Kitron U (2009). A new, cost-effective, battery-powered aspirator for adult mosquito collections. J Med Entomol.

[36] Odiere M, Bayoh MN, Gimnig J, Vulule J, Irungu L, Walker E (2007). Sampling outdoor, resting *Anopheles gambiae* and other mosquitoes (Diptera: Culicidae) in western Kenya with clay pots. J Med Entomol.

[37] Scott JA, Brogdon WG, Collins FH (1993). Identification of single specimens of the *Anopheles gambiae* complex by the polymerase chain reaction. Am J Trop Med Hyg.

[38] Koekemoer LL, Kamau L, Hunt RH, Coetzee M (2002). A cocktail polymerase chain reaction assay to identify members of the *Anopheles funestus* (Diptera: Culicidae) group. Am J Trop Med Hyg.

[39] Njie M, Dilger E, Lindsay SW, Kirby MJ (2009). Importance of eaves to house entry by anopheline, but not culicine, mosquitoes. J Med Entomol.

[40] Geier M, Bosch OJ, Boeckh J (1999). Influence of odour plume structure on upwind flight of mosquitoes towards hosts. J Exp Biol.

[41] Tusting LS, Willey B, Lines J (2016). Building malaria out: improving health in the home. Malar J.

[42] Furnival-Adams J, Olanga EA, Napier M, Garner P (2020). House modifications for preventing malaria. Cochrane Database Syst Rev.

[43] Ogoma SB, Lweitoijera DW, Ngonyani H, Furer B, Russell TL, Mukabana WR (2010). Screening mosquito house entry points as a potential method for integrated control of endophagic filariasis, arbovirus and malaria vectors. PLoS Negl Trop Dis.

[44] Githeko AK, Service MW, Mbogo CM, Atieli FK, Juma FO (1994). Origin of blood meals in indoor and outdoor resting malaria vectors in western Kenya. Acta Trop.

[45] Githeko AK, Adungo NI, Karanja DM, Hawley WA, Vulule JM, Seroney IK (1996). Some observations on the biting behavior of *Anopheles gambiae* s.s., *Anopheles arabiensis*, and *Anopheles funestus* and their implications for malaria control. Exp Parasitol.

[46] Bayoh MN, Mathias DK, Odiere MR, Mutuku FM, Kamau L, Gimnig JE (2010). *Anopheles gambiae*: historical population decline associated with regional distribution of insecticide-treated bed nets in western Nyanza Province, Kenya. Malar J.

[47] McCann RS, Ochomo E, Bayoh MN, Vulule JM, Hamel MJ, Gimnig JE (2014). Reemergence of Anopheles funestus as a vector of *Plasmodium falciparum* in western Kenya after long-term implementation of insecticide-treated bed nets. Am J Trop Med Hyg.

[48] Jawara M, Jatta E, Bell D, Burkot TR, Bradley J, Hunt V (2018). New prototype screened doors and windows for excluding mosquitoes from houses: a pilot study in rural Gambia. Am J Trop Med Hyg.

[49] Shenton FC, Jawara M, Carrasco-Tenezaca M, Knudsen J, D'Alessandro U, Lindsay SW (2022). The durability, functionality and acceptability of novel screened doors and windows after 4 years of use in a Gambian village: a cross-sectional survey. Malar J.

[50] Alberto DAS, Rusch C, Zhan Y, Straw AD, Montell C, Riffell JA (2022). The olfactory gating of visual preferences to human skin and visible spectra in mosquitoes. Nat Commun.

[51] Lindsay SW, Jawara M, Mwesigwa J, Achan J, Bayoh N, Bradley J (2019). Reduced mosquito survival in metal-roof houses may contribute to a decline in malaria transmission in sub-Saharan Africa. Sci Rep.

[52] Gimnig JE, Kolczak MS, Hightower AW, Vulule JM, Schoute E, Kamau L (2003). Effect of permethrin-treated bed nets on the spatial distribution of malaria vectors in western Kenya. Am J Trop Med Hyg.

[53] https://www.againstmalaria.com/dollarspernet.aspx. Why US$2.00 per net? : Against Malaria Foundation; 2022: https://www.againstmalaria.com/dollarspernet.aspx.

[54] Alonso S, Chaccour CJ, Wagman J, Candrinho B, Muthoni R, Saifodine A (2021). Cost and cost-effectiveness of indoor residual spraying with pirimiphos-methyl in a high malaria transmission district of Mozambique with high access to standard insecticide-treated nets. Malar J.

[55] AfDB, OECD, UND. African Economic Outlook 2017 Africa Development Bank; 2017.

